# Occurrence of potentially pathogenic nontuberculous mycobacteria in Mexican household potable water: a pilot study

**DOI:** 10.1186/1756-0500-6-531

**Published:** 2013-12-11

**Authors:** Iza Perez-Martinez, Diana A Aguilar-Ayala, Elizabeth Fernandez-Rendon, Alma K Carrillo-Sanchez, Addy C Helguera-Repetto, Sandra Rivera-Gutierrez, Teresa Estrada-Garcia, Jorge F Cerna-Cortes, Jorge A Gonzalez-y-Merchand

**Affiliations:** 1 Departamento de Microbiologia, Escuela Nacional de Ciencias Biologicas-Instituto Politecnico Nacional, Prolongacion Carpio y Plan de Ayala S/N, Col. Casco de Santo Tomas, Delegacion Miguel Hidalgo, Mexico, D.F. CP 11340, Mexico; 2 Departamento de Biomedicina Molecular, CINVESTAV-IPN, Av. IPN 2508, Zacatenco, Mexico, DF CP 07360, Mexico

**Keywords:** Household water microbiological quality, Mycobacteria in potable water, Nontuberculous mycobacteria (NTM), *Mycobacterium mucogenicum*

## Abstract

**Background:**

Nontuberculous mycobacteria (NTM) are environmental opportunistic pathogens found in natural and human-engineered waters, including drinking water distribution systems and household plumbing. This pilot study examined the frequency of occurrence of NTM in household potable water samples in Mexico City. Potable water samples were collected from the “main house faucet” and kitchen faucet. The presence of aerobic-mesophilic bacteria (AMB), total coliforms (TC), fecal coliforms (FC) and NTM species were determined. Mycobacteria species were identified by PCR restriction enzyme pattern analysis (PRA) of the 65-kDa heat shock protein gene (*hsp65*) and sequencing of the hypervariable region 2 (V2) of the 16S rRNA gene and of the *rpo*B gene.

**Results:**

AMB (<100 CFU/ml) were present in 118 out of 120 samples; only two samples were outside guidelines ranges (>100 CFU/ml). TC and FC were detected in four and one samples, respectively. NTM species were recovered from 16% samples (19/120) and included *M. mucogenicum* (nine), *M. porcinum* (three), *M. avium* (three), *M. gordonae* (one), *M. cosmeticum* (one), *M. fortuitum* (one), and *Mycobacterium* sp (one). All household water samples that contained NTM complied with the standards required to grade the water as “good quality” potable water.

**Conclusion:**

Household potable water may be a potential source of NTM infection in Mexico City.

## Background

Since the discovery of *Mycobacterium leprae* by Armauer Hansen in 1873 and *M. tuberculosis* by Robert Koch in 1883, more than one century ago, 165 mycobacterial species have been validly described [1]. The majority of mycobacteria species belong to the nontuberculous mycobacteria (NTM) group and most of them can be isolated from the environment [2]; they are opportunistic pathogens that may cause life-threatening infections in humans [2]. In recent years, morbidity and mortality associated to NTM illness has increased in both immunocompetent and immunocompromised subjects worldwide [3-5]. Some NTM species can cause pulmonary disease, affect the skin, lymphatic nodes and gastrointestinal tract and can produce disseminated disease in severely immunocompromised individuals [6].

NTM are resilient bacteria that grow in virtually any environment, including water bodies where competing microorganisms are eliminated, such as in chlorinated water [7,8]. The growth of NTM in biofilms may lead to their dissemination into bulk water; hence, people may be exposed to these mycobacteria when drinking, bathing, and showering (inhalation of aerosols). NTM species have been isolated from hospital water samples including tap water, shower water and aerosolized shower mist [9,10] and NTM presence in hospital water samples has been linked to nosocomial outbreaks [9]. A recent study using DNA fingerprint analysis revealed that household water was the source of mycobacterial infection in patients with NTM disease [11]. The aim of this pilot study, therefore, was to determine the frequency of occurrence of NTM in potable water samples from homes in Mexico City, taking as the framework of reference, the microbiological quality of those samples.

## Methods

### Area of study and water sample collection

**Figure 1:**
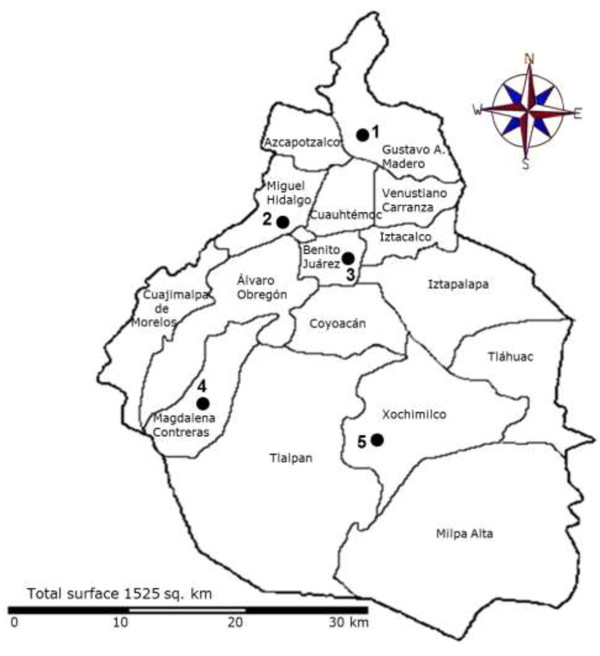
**Household sampling sites (●), in Mexico City.** Gustavo A. Madero borough (site 1), Miguel Hidalgo borough (site 2), Benito Juárez borough (site 3), Magdalena Contreras borough (site 4), Xochimilco borough (site 5). Sites 1 and 5 received groundwater from wells and sites 2, 3 and 4 received surface water from rivers (see also Table 1).

The selected area of study was Mexico City; the water distributed in this area includes groundwater (pumped from wells) and surface water from the Cutzamala and Magdalena rivers. As shown in Figure 1, five households were randomly selected, two of which are supplied by groundwater and the remaining 3, from surface water. In Mexico City, the surface water treatment process consists of chlorination, coagulation, and flocculation combined with rapid sand filtration. Well water is treated by chlorination/dechlorination, granular active carbon filtration, ion exchange filtration, reverse osmosis and chlorination. The definition of what constitutes potable water in Mexico City is mentioned in Additional file 1. From November 2008 to October 2009 a total of 120 potable water samples were collected from both the “main house faucet” and kitchen faucet of the household. Faucets were externally disinfected prior to the water collection. All samples were collected once or twice from each house every month, from November to October. For mycobacterial isolation from each faucet, 1 liter of water was collected in a sterile polypropylene bag (NASCO, Fort Atkinson, WI, USA) containing 0.5 ml of 10% sodium thiosulphate (Na_2_S_2_O_3_) to neutralize any free chlorine present in the sample upon collection [12]. For chemical and microbiological quality analyses, another liter of water was collected in a sterile plastic tube that did not contain Na_2_S_2_O_3_. Samples were processed within 2 h after collection.

### Chemical and microbiological analysis

The pH and chlorine residual concentrations of all water samples were determined by using pH test strips and the orthotolidine technique, respectively, as recommended by Standard American Public Health Association Procedures [13]. The presence of aerobic-mesophilic bacteria (AMB), total coliforms (TC) and fecal coliforms (FC) in all water samples was assessed by standard methods [13].

### Isolation and identification of mycobacteria

One liter of water was filtered through a membrane (pore size: 0.45 μm; Millipore, Billerica, MA, USA). The membrane was cut in two pieces; each piece was put on to Lowenstein-Jensen slopes (BBL) and incubated at 35°C. Tubes were examined daily for the first eight days and thereafter once a week for two months. Once the bacterial growth had been observed on the Lowenstein-Jensen slopes, the identification of acid-fast bacilli was carried out by Ziehl-Neelsen stain. Acid-fast bacilli were subcultured on Middlebrock 7H10 agar at 35°C, labeled by sampling location and with a consecutive number.

Strains belonging to the genus *Mycobacterium* and to the *M. tuberculosis* complex were identified by two PCR assays previously described [14,15]. Therefore, by exclusion, mycobacteria strains that did not belong to the *M. tuberculosis* complex were considered to be NTM. Briefly, 3 μl aliquots of bacterial lysates were subjected to amplification, using a standard *Taq* polymerase (Life Technologies, Rockville, MD) in a total volume of 50 μl of PCR mixture; RAC1 and RAC8*,* and MTB-F and MTB-R primers [15] were used for identification of the *Mycobacterium* genus and of the strains belonging to the *M. tuberculosis* complex, respectively. A fragment using the primer combination of RAC1 and RAC8 was amplified; this amplicon contains the last 99 codons of the *mur*A gene, the promoter region of the *rrn*A operon and 360 nucleotides from the 5′ end of the 16S rRNA gene. As shown by Perez-Martinez *et al.*[14], the amplicon size varies within the mycobacteria species from 934 to 1300 bp. MTB-F/MTB-R primers amplified a gene fragment coding for the last five codons of *mur*A gene, the promoter region of the *rrn*A operon and the 5′end of the 16S rRNA; a 488 bp fragment characteristic only of *M. tuberculosis* complex members is amplified.

NTM were identified by three methods: (i) PCR restriction enzyme pattern analysis (PRA) of the 65-kDa heat shock protein gene (*hsp65*), as described by Telenti *et al*. [16]*;* (ii) sequencing of the hypervariable region 2 (V2) of the 16S rRNA gene [17]; and (iii) sequencing of the *rpo*B gene [18]. Mycobacterial PRA was performed by PCR amplification of a 439-bp fragment of the *hsp*65 gene by using primers Tb11 and Tb12 [16]. PCR products were visualized by agarose gel electrophoresis, after which 10 μl volumes of each PCR product were digested in two separate reactions with two restriction enzymes, BstEII (New England BioLabs) and HaeIII (Invitrogen). Digested products were then analyzed using the Agilent 2100 bioanalyzer. DNA 1000 LabChips (Agilent) were used according to manufacturer’s protocol. PRA results were interpreted with the algorithm described by Telenti *et al*. [16], which is available on the PRA database [19]. Identification of the mycobacterial species was obtained by automatized sequence of the hypervariable region 2 (V2) of the 16S rRNA gene and of the *rpo*B gene. The amplification of the 16S rRNA gene was performed using the RAC1 and RAC8 primers [15]. For the amplification of the *rpo*B gene, the MF and MR primers were used to obtain a product of 342 pb, useful for the identification of mycobacterial species [18]. Both products of PCR were sequenced using the RAC8 [15] and MF [18] primers, respectively, and the big dye terminator ready reaction kit (Perkin-Elmer, Inc, Wellesley, MA). The sequences were analyzed by ABI PRISM 310 genetic analyzer system (Perkin- Elmer). Nucleotide sequences were compared to known sequences in the GenBank database by using the Blastn algorithm. Species identifications were based on the 100% similarity cut-off for the 16S rRNA gene and ≥97% for the *rpo*B gene.

## Results

A total of 120 potable water samples were collected from 5 households, which were considered to be representative homes from the studied area because they are distributed throughout the city, covering northern, central, and southern areas of the most populated areas of Mexico City. These were also chosen in this way because two of them receive drinking water from wells and three from rivers (Figure 1). Two households provided 60 samples of groundwater as their supply; the remaining 60 samples came from 3 houses supplied by surface water (Table 1). Water samples had a pH range of 6.8 to 7.8 and a chlorine concentration range from 0.2 to 1.5 ppm: all samples were thus found to be within the range of standards set up by Mexico’s Official Guidelines for potable water [20] for both chlorine concentrations (0.2 - 1.5 ppm) and pH (6.5 - 8.5).

**Table 1:** Household water type and source and number of NTM positive samples and species identified

**Water type and source**	**Household**	**Sampling sites**	**Number of samples collected**	**Number of positive samples (%)**	**(Number) NTM identified by 16S rRNA, *hsp*** **65 and *rpo*** **B genes**
	**Region**				
	**(see Figure**1**)**				
**Groundwater**					
Well	1	MHF^*^	16	4 (25)	(3) *M. mucogenicum*, (1) *M. gordonae*
		KF^**^	16	4 (25)	(4) *M. mucogenicum*
Well	5	MHF	14	4 (29)	(3) *M. porcinum*, (1) *Mycobacterium sp*
		KF	14	0 (0)	
**Surface water**					
Cutzamala system	2	MHF	10	2 (20)	(1) *M. avium,* (1) *M. mucogenicum*
		KF	10	0 (0)	
Cutzamala system	3	MHF	10	1 (10)	(1) *M. avium*
		KF	10	1 (10)	(1) *M. cosmeticum*
Magdalena River	4	MHF	10	1 (10)	(1) *M. fortuitum*
		KF	10	2 (20)	(1) *M. avium,* (1) *M. mucogenicum*

^*^ “main house faucet”.

^**^ kitchen faucet.

Microbiological analysis showed that AMB (<100 CFU/ml) were present in 118 of the 120 samples and only 2 samples were outside guidelines ranges (>100 CFU/ml). The average value of CFU/ml corresponding to these AMB per household per month varied as follows: 12 CFU/ml for household 3, 15 CFU/ml for household 4, 38 CFU/ml for household 1, 60 CFU/ml for household 5 and 87 CFU/ml for household 2. According to these results and the data shown in Table 1, there was no correlation between the average number of CFU/ml of AMB and the presence of NTM in the same sample, e.g. household 2 contained the highest amount of AMB/ml (87), but NTM were isolated in two samples only. In stark contrast, household 1, with half of the amount of AMB/ml, contained eight positive samples for NTM, and for this reason, AMB data cannot be considered as an indication for the presence of NTM in the drinking water.

Regarding fecal contamination indicators, while TC were detected in household 1 (February), 2 (April and June) and 4 (August), FC were only detected in one sample (household 2, in June). These four samples presented a range of MPN/100 ml from 2.2 to >16. None of these water samples contained NTM.

**Figure 2:**
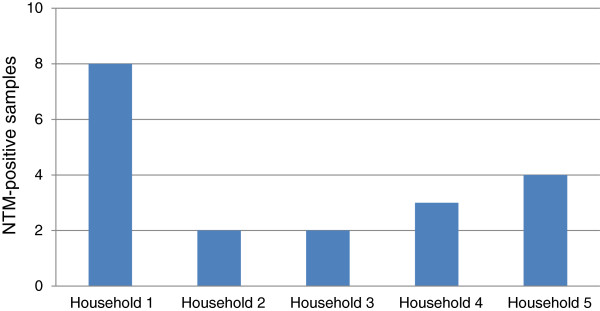
Number of positive-NTM samples per household.

Mycobacteria-like microorganisms were isolated in 19 (16%) of the 120 samples analyzed (Figure 2 and Table 1). Although household 1 showed more positive samples (eight) than others, NTM were observed at all of them (Figure 2). The 19 isolates identified as *Mycobacterium* were identified as follows: 9 (47%) *M. mucogenicum*, 3 (16%) *M. porcinum*, 3 (16%) *M. avium*, 1 (5%) *M. gordonae*, 1 (5%) *M. cosmeticum*, 1 (5%) *M. fortuitum* and 1 (5%) *Mycobacterium* sp, none belonging to MTC (Table 1). Most *M. mucogenicum* (78%) and all *M. porcinum* and *M. gordonae* strains were isolated from households that were supplied by groundwater samples, while *M. avium* strains were only isolated from surface water samples. Interestingly, there is one report where *M. gordonae* was also isolated from a groundwater sample in Mexico City [21]. Comparison of the number and species of NTM isolated from the households’ “main faucets” and kitchen faucets, showed no significant differences (Table 1).

**Figure 3:**
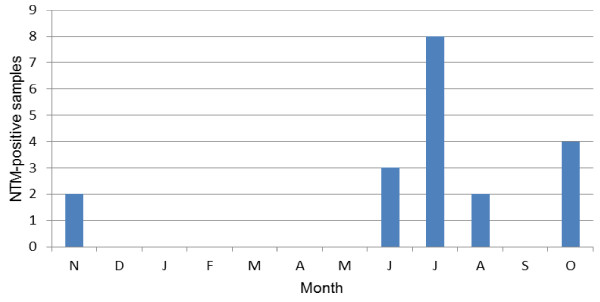
Number of positive-NTM household water samples per month during November 2008 to October 2009.

As shown in Figure 3, mycobacteria species were isolated only during summer and autumn: 68.4% (13/19) of the strains were isolated in summer, which is the rainy season in Mexico City, and the remaining 31.6% were isolated in autumn.

## Discussion

In this study, we combined culture and molecular methods for detection and identification of NTM in drinking water samples which were directly collected from households from various areas of Mexico City. The chosen methodology was based on the comparison study reported by Thomson *et al*. [12], mainly, using their recommendations: use of filtration as a simpler and more time efficient process to isolate mycobacteria; the presence of contaminants that does not affect mycobacteria isolation capability; use of different incubation temperatures or different solid media for long periods that does not affect the isolation; and use of cetylpyridinium chloride that controls contamination but also reduces mycobacterial growth.

Despite chlorination being detected in all water samples, and considering the Mexican standards for water intended for human use and consumption based on ≤2 MPN/100 ml of CT and the absence of FC [20], the study shows that only 4 samples did not meet these standards. These results show that the standard levels of chlorine (0.2 – 1.5 ppm) maintained in the distribution system as it reaches the customer’s tap are not sufficient to inactivate all microorganisms that may have entered the pipelines. There exists the possibility that faucets have been contaminated by people living in the home, so that TC and/or FC were already inside the faucet, rather than coming from the drinking water distribution system itself. The particular value of maintaining chlorine residual is the prevention of growth of slime in the system and more importantly, to be a security compound to indicate whether recontamination (within the pipelines) may have occurred. Our results are consistent with those reported by Mazari-Hiriart *et al.*[22] and Villarruel-Lopez *et al*. [23], who reported the presence of AMB, CT and FC in several drinking water samples in Mexico City. However, most of the household water samples complied with the guidelines for “good quality” potable water. It is important to consider that, in spite of having processed the samples within 2 h of collection, the amounts of AMB, CT and FC found in our study might be underestimated since there was still some residual chlorine presented in the collected 1 liter water samples that did not contain Na_2_S_2_O_3_.

The frequency of NTM recovery from household water was 16%, similar to that found in a recent study of potable water in Uganda [24] and in another from the USA [11]. In spite of that, our percentage figure may represent an underestimation of the NTM presence in these samples because some reports [25,26] have demonstrated that there are NTM that could be viable but are not able to be cultured, along with some others that resist culture because growth requirements for all NTM are not yet known.

A high percentage of NTM species have been identified from drinking water distribution systems in Germany (57%), France (72%), Finland (between 30-80%), and Australia (62%) and yet the chemical and microbiological qualities of these samples were not reported [25,27-29]. None of these previous works took water samples directly from the faucets inside the homes. An additional study in the USA, involving different kinds of drinking water (cisterns, bottled water and ice samples, among others), reported a percentage of NTM occurrence of 33%, being *M. mucogenicum* the most frequent species isolated [30]. This particular species was also the most frequent isolate in our study (see Table 1).

Comparing several studies [24,27,29,30] carried out seeking NTM in drinking water with ours, we observed that while there are some species repeatedly found in all potable water samples, (such as *M. gordonae* and *M. fortuitum*), others (as *M. porcinum* and *M. cosmeticum*) were only found in our water samples. Besides, *M. avium* in particular has been frequently reported from drinking water in the USA [7,11,30]. It would be advisable to carry out further studies about isolation of NTM from drinking water in other countries and in all states of Mexico, in order to confirm the possibility of the existence of certain species of NTM, depending of the geographical part of the world.

Some of the NTM identified in our study included species that have been frequently associated with human illness in other countries, such as: *M. avium*, *M. fortuitum*, and *M. mucogenicum*[31]. In Mexico City, the prevalence of NTM infections is poorly known and few studies have been published, among them, Lopez-Alvarez *et al.*[32] reported in 2010 that 15% of mycobacterial strains isolated from 67 HIV patients belonged to NTM (10 strains were identified as *M. avium* and 1 as *M. intracellulare*). In another recent study, Cortes-Torres *et al.*[33] in 2013 reported that 37% of 96 patients in a Mexico City Hospital, with various immunodeficiencies, presented various strains of NTM, of which 23 were *M. avium*, 9 *M. simiae*, 2 *M. gordonae* and 2 *M. kansasii*. Although no combined conclusion could be reached between these several findings and ours, further studies of occurrence of NTM from different sources in our country should be carried out, in order to confirm that NTM isolated from drinking water are the same as those isolated from patients.

According to our results (Table 1) of the NTM species isolated from groundwater and surface water samples, and taking into consideration that the number of NTM isolates that is too low in our study to be able to draw general conclusions, we can suggest a possible correlation between an aquatic environment from which mycobacteria are isolated and the particular species of NTM that is found in such habitat. This should be further studied in order to confirm our hypothesis.

NTM cell surface hydrophobicity allows the attachment of these microorganisms to surfaces and favors their capacity for biofilm formation, particularly under low-nutrient conditions [34,35]. Therefore, it seems that NTM are not transient contaminants of drinking water distribution systems but rather that they grow and persist in household plumbing [7,11].

The results of our analysis by seasons suggest a direct correlation between a steady increase in the amount of recovered NTM from the environment and average rainfall and temperature, as do those carried out in Uganda and the Netherlands [24,26]. It is noteworthy that human NTM illness is more frequently reported during the summer and autumn seasons [36].

This study allowed us to apply a useful methodology for NTM identification from household potable water in Mexico City, which translates into the need for a larger future study of households in Mexico City in order to confirm NTM species distribution by water source, to quantify their concentration, and to identify the contributing factors that permit the persistence of such microorganisms in water distribution systems.

## Conclusions

This pilot study recovered some potentially pathogenic NTM species from Mexican household potable water, even though the water complied with the guidelines established to qualify as “good quality” potable water. Measures to diminish or eliminate NTM strains from household water might be advisable, at least on an individual basis, for immunosuppressed patients at home.

## Competing interests

The authors declare that they have no competing interests.

## Authors’ contributions

Conception and design of the experiments: EF-R, JFC-C, JAG-y-M. Performance of the experiments: IP-M, DAA-A, AKC-S, ACH-R, SR-G. Data analysis: IP-M, DAA-A, JFC-C, JAG-y-M. Writing of the paper: JFC-C, TEG, JAG-y-M. All Authors read and approved the final manuscript.

## Supplementary Material

Additional file 1 Definitions. Click here for file
