# Supplementary material for: Occurrence of potentially pathogenic nontuberculous mycobacteria in Mexican household potable water: a pilot study
Source: BMC Res Notes. 2013 Dec 11;6:531. doi: 10.1186/1756-0500-6-531 (PMC3874667; doi:10.1186/1756-0500-6-531)
Supplement: Additional file 1 — Definitions. [file 1756-0500-6-531-S1.docx]

**Definitions.**

The term ‘**potable**’ in Mexico means drinkable or fresh water drawn from the main water supply, which can goes directly to the kitchen taps, showers, baths and lavatories, or which can be stored within cisterns to be used without further treatment and from there also goes to the kitchen and bathrooms. In Mexico all homes have a “main household faucet” to which the water line from the water distribution system runs into the home.

The **Metropolitan Area of Mexico City** has a population of more than 21 million, is the third [largest metropolitan area](http://en.wikipedia.org/wiki/List_of_metropolitan_areas_by_population) in the world and the [most populous metropolitan city in the Americas](http://en.wikipedia.org/wiki/List_of_metropolitan_areas_in_the_Americas_by_population). This region is constituted by Mexico City and adjacent [municipalities](http://en.wikipedia.org/wiki/Municipalities_of_Mexico) of the States of [Mexico](http://en.wikipedia.org/wiki/State_of_Mexico) and [Hidalgo](http://en.wikipedia.org/wiki/Hidalgo_(state)). Mexico City has almost 9 million inhabitants and is divided into 16 "delegaciones" or boroughs (Figure 1).

The **Cutzamala system** is the second major source of water for the Metropolitan Area of Mexico City. It supplies water to the north of Mexico City and to the State of Mexico. In 1976, the project known as Cutzamala system (“sistema Cutzamala”) was planned to supply water to the Metropolitan Area of Mexico from both, the Cutzamala and the Lerma-Balsas Rivers (in the State of Mexico), in order to reduce the over-exploitation of the aquifer of the Valley of Mexico.
